# The Modified A-DIVA Scale as a Predictive Tool for Prospective Identification of Adult Patients at Risk of a Difficult Intravenous Access: A Multicenter Validation Study

**DOI:** 10.3390/jcm8020144

**Published:** 2019-01-26

**Authors:** Fredericus H. J. van Loon, Loes W. E. van Hooff, Hans D. de Boer, Seppe S. H. A. Koopman, Marc P. Buise, Hendrikus H. M. Korsten, Angelique T. M. Dierick-van Daele, Arthur R. A. Bouwman

**Affiliations:** 1 Department of Technical and Anesthesia Nursing Sciences, Fontys University of Applied Sciences, 5631 Eindhoven, The Netherlands; 2 Department of Anesthesiology, Pain Medicine and Intensive Care, Catharina Hospital, 5623 Eindhoven, The Netherlands; marc.buise@catharinaziekenhuis.nl (M.P.B.); erik.korsten@catharinaziekenhuis.nl (H.H.M.K.); arthur.bouwman@catharinaziekenhuis.nl (A.R.A.B.); 3 Emergency Department, Catharina Hospital, 5623 Eindhoven, The Netherlands; loes.v.hooff@catharinaziekenhuis.nl; 4 Department of Anesthesiology, Pain Medicine and Procedural Sedation and Analgesia, Martini General Hospital, 9728 Groningen, The Netherlands; hd.de.boer@mzh.nl; 5 Department of Anesthesiology, Maasstad Hospital, 3079 Rotterdam, The Netherlands; koopmanj@maasstadziekenhuis.nl; 6 Department of Signal Processing Systems and Electrical Engineering, TU/e University of Technology, 5612 Eindhoven, The Netherlands; 7 Department of People and Health Sciences, Fontys University of Applied Sciences, 5631 Eindhoven, The Netherlands; angelique.dierick@catharinaziekenhuis.nl; 8 Department of Research and Education, Catharina Hospital, 5623 Eindhoven, The Netherlands

**Keywords:** difficult intravenous access, vascular access devices, catheterization, peripheral, decision-making, intravenous therapy, logistic models, risk factors

## Abstract

Peripheral intravenous cannulation is the most common invasive hospital procedure but is associated with a high failure rate. This study aimed to improve the A-DIVA scale (Adult Difficult Intra Venous Access Scale) by external validation, to predict the likelihood of difficult intravenous access in adults. This multicenter study was carried out throughout five hospitals in the Netherlands. Adult participants were included, regardless of their indication for intravenous access, demographics, and medical history. The main outcome variable was defined as failed peripheral intravenous cannulation on the first attempt. A total of 3587 participants was included in this study. The first attempt success rate was 81%. Finally, five variables were included in the prediction model: a history of difficult intravenous cannulation, a difficult intravenous access as expected by the practitioner, the inability to detect a dilated vein by palpating and/or visualizing the extremity, and a diameter of the selected vein less than 3 millimeters. Based on a participant’s individual score on the A-DIVA scale, they were classified into either a low, moderate, or high-risk group. A higher score on the A-DIVA scale indicates a higher risk of difficult intravenous access. The five-variable additive A-DIVA scale is a reliable and generalizable predictive scale to identify patients at risk of difficult intravenous access.

## 1. Introduction

In modern day healthcare, hospital inpatients commonly require the insertion of vascular access devices, with an estimated prevalence of peripheral intravenous cannulation of up to 85% [1,2,3]. Hospitalized patients need a peripheral intravenous catheter to administer drugs, fluids, and blood products during their admission, and successful first-attempt peripheral intravenous cannulation ensures prompt administration of these drugs and fluids intravenously [1,2,3,4,5]. Although peripheral intravenous cannulation is the most common invasive hospital procedure performed worldwide, it is associated with an unacceptable high overall failure rate [5,6]. An unsuccessful attempt of intravenous cannulation poses a burden to patients, caregivers, and the healthcare system, because of an increasing number of painful and stressful punctures, nursing and medical workload, and catheter-related infections and phlebitis [5,7,8].

Intravenous therapy can be defined as any form of treatment in which access to a vein is necessary [1,6,8]. The procedure of peripheral intravenous cannulation is performed routinely in daily clinical practice, while the first attempt to obtain an intravenous access is not successful in every patient [2,4,5,8,9,10,11]. A recently published systematic review and meta-analyses reported a failure rate of up to 30% on the first attempt of peripheral intravenous cannulation with the conventional approach [6]. This conventional approach of peripheral intravenous cannulation involves visual inspection and palpation of the extremity to locate a vein, followed by a needle puncture and catheter insertion [8,12]. Notwithstanding, intravenous access can be difficult to obtain, especially in patients with a lack of visual or palpable apparent veins, smaller veins, and in patients with a known history of a difficult intravenous access [11]. 

Although advances have been made by recent research, focus seems mainly to be on the procedure of peripheral intravenous cannulation itself. In routine clinical practice, a difficult intravenous access is identified after performing multiple attempts to insert an intravenous catheter. Identification of the presence of a difficult intravenous access prospectively, however, can possibly lower the incidence of a failed first attempt of inserting a peripheral intravenous catheter and improve patients’ outcomes [6,11]. The A-DIVA scale (Adult Difficult IntraVenous Access Scale) is a recently created predictive scale to identify patients with a difficult intravenous access [11]. This additive A-DIVA scale implies the prediction of the likelihood of a difficult intravenous access in adult patients prospectively based on clinical observations, and consisted of five variables, including an inability to detect a vein suitable for cannulation by palpating and/or visualizing the extremity, a vein diameter of two millimeters or less, a known history of a difficult intravenous access, and an emergency indication for surgery [11]. Predictive models have been developed and widely used in modern, evidence-based medicine to estimate the probability of the presence of a particular event in an individual patient, which led to a reduction in healthcare costs, due to greater hospital efficiency by using staff time and equipment effectively [13,14,15,16].

The previous described A-DIVA scale was developed in a cohort of surgical patients, in which anesthesiologists and nurse anesthetists inserted a short peripheral intravenous catheter into the upper extremity [11]. However, before considering whether to use a clinical predictive scale, it is essential that its predictive performance is evaluated empirically [15,16]. Both external and internal validation techniques have been developed to evaluate predictive models, with external validation being the most stringent test [17,18]. External validation explores differences in characteristics of cohorts and examines how well a model performs in other datasets than in which the prediction model was developed [17,18]. On that account, to improve clinical usability and to confirm the A-DIVA scale to be generalizable to all hospitalized patients, it is essential to evaluate its performance in an external cohort [17].

The present study focuses on risk factors for failure upon peripheral intravenous cannulation and aims to improve the performance of the A-DIVA scale by creating a predictive scale that is externally validated and applicable to the total hospitalized population. To this end, an external validation study was performed including participants from different units of multiple Dutch hospitals.

## 2. Materials and Methods

### 2.1. Design and Setting

This multicenter and multidisciplinary cross-sectional study was approved by the Institutional Ethics Committee (Catharina Hospital, ref: 2015-21) and registered in the Dutch Trial Register (ref: NTR5846). Written informed consent was obtained from all participants, who were studied between January 2017 and May 2018 throughout five hospitals in The Netherlands: Catharina Hospital, Eindhoven; Martini Hospital, Groningen; Zuyderland Hospital, Heerlen; Saint Anna Hospital, Geldrop; and Maasstad Hospital, Rotterdam.

### 2.2. Participants

Participants, both inpatients and outpatients, were recruited from different units of the hospitals mentioned before, including the preoperative holding area of the theatre complex, the emergency department, and the labor ward. All participants were over 18 years of age, and asked for participation regardless their American Society of Anesthesiology (ASA) physical status, demographics, and medical history. Inclusion of participants on this low threshold, however, resulted in the most real reflection of the actual population of inpatients. Participants were excluded if they did not provide written informed consent, did not understand or answer the questionnaire, when intravenous access had already been gained, and in cases of protocol violation. 

### 2.3. Sample Size Calculation

The sample of participants was randomly divided into two subsets: a development cohort and an evaluation (validation) cohort. A non-probability, consecutive sampling technique was used throughout the inclusion period, in which every participant meeting the in- and exclusion criteria was included until the required sample size in both cohorts was achieved. The development cohort existed of one third (33%) of the total sample of participants in this study. Sample size calculation was based on the first-attempt failure rate of 17%, as recorded in the previously performed A-DIVA study [11]. At least 5 participants with a present event needed to be enrolled for each predictor in the univariate logistic model [19]. Furthermore, at least 10 participants with an event were needed to be included for each predictor in the multivariate logistic model [19]. A convenience and quota sampling of at least 1100 participants therefore had to be included in the development cohort. The other two thirds (66%) of participants were included in the evaluation cohort, which consisted of 2200 participants, to ensure a balanced distribution across the desired variables. 

### 2.4. Procedure

Practitioners, both nurses and physicians from any medical specialism or unit who were familiar with the study protocol, routinely obtained peripheral intravenous access. All participating practitioners in this study were experienced in peripheral intravenous cannulation, based on a minimum experience of one year in clinical practice. Residents and practitioners in training were excluded from participating in this study. A short peripheral intravenous catheter was inserted into the upper extremity, and veins on the dorsal and ventral surfaces of the upper extremity were considered for peripheral cannulation, including the metacarpal, cephalic, basilic, and median veins [12]. Intravenous cannulation was performed according to practice guidelines, by applying the traditional approach of palpating and visualizing the extremity [8,12]. The size of the inserted intravenous catheters ranged between 14 to 22 gauge, depending on the clinical situation [8,12].

### 2.5. Primary Outcome

The primary outcome variable was defined as failed peripheral intravenous cannulation on the first attempt. Intravenous cannulation was considered successful if the practitioner was able to inject a saline flush without signs of infiltration [8,12]. We determined one attempt as a percutaneous needle puncture, regardless of the amount of subcutaneous exploration from the single puncture site [12]. After a failed attempt, a new attempt was executed by firstly localizing a vein, followed by a new percutaneous puncture [12]. After two failed attempts by one practitioner, the following attempts were performed by another practitioner [8,12].

### 2.6. Predictors

Predictors for difficult intravenous access were selected from clinical observations, literature search in recent publications, and by expert opinions in a focus meeting discussion group. Baseline demographic data (gender, age, weight, length, ASA physical status, a participant’s skin shade classified on a three-point scale based on race and origin, and the dominant side of a participant), participants’ social behavior (active smoking, intravenous drug abuse, and alcohol abuse), and the medical history (cardiac diseases, pulmonary diseases, vascular diseases, a history of chemotherapy treatment, haematological status, the use of any medications, and hypovolemia based on haemodialysis or dehydration) were provided by the local institutional clinical registry, which contains information in a computerized database. Procedure-related data included whether or not the first attempt was successful and the number of attempts needed for successful intravenous cannulation, side of cannulation (left or right), place of cannulation on the extremity (dorsal side of the hand, lower arm, elbow crease, or upper arm), size of the intravenous catheter, if it was difficult to achieve an intravenous access in the past (was established by asking the participant), a practitioner’s expectation of difficult intravenous cannulation prior to the puncture based on his perception and experience, whether or not a suitable vein could be identified by palpating and/or visualizing the upper extremity, diameter of the vein measured in millimetres after applying a tourniquet by placing a ruler on the vein, a participants pain score after cannulation on an eleven-points NRS scale (Numeric Rating Scale) (0 means no pain at all, whereas a score of 10 means the most worst imaginable pain), and characteristics regarding the practitioner performing the procedure (profession of the practitioner), and were prospectively collected by the practitioner who performed the procedure. Study related registrations were performed on standardized data abstraction forms. Both patients and practitioners were blinded to the outcome of interest and were not aware of the purpose of this research project. 

### 2.7. Statistical Analysis

The Kolmogorov-Smirnov test assessed the normality assumption for continuous variables, which were expressed as mean and standard deviation (SD), those without as median and interquartile range (IQR). Discrete variables were expressed as frequencies with percentages. Comparison of variables was executed using the Chi-squared test or Fisher’s exact test for discrete variables, and the Student’s *t*-test or the Mann-Whitney *U*-test for continuous variables, as appropriate. 

To create the modified A-DIVA scale, a univariate logistic regression analysis was performed to obtain the odds ratio for failed intravenous cannulation on the first attempt [18,20]. Thereafter, a multivariate logistic analysis was performed including the variables with a *p* < 0.05 from the univariate analysis for examining their independent association with the primary outcome [14,18,20]. Variables were removed from this model using a backward elimination process, with the removal criteria set at *p* < 0.001 [14,20]. The definitive predictive scale was constructed by including significant variables from the multivariate logistic analysis. A predictive scale has to be effective and efficient in its use, and clinical usability will be improved by including the smallest set of variables in the final model. Hence, the removal criteria at *p* < 0.0001 in the multivariate logistic regression will lead to elimination of questionable variables from the model, to comply with this assumption. The effect size of all independent predictors was reported with adjusted odds ratios (OR) and 95% confidence intervals (95% CI). The additive A-DIVA scale was created afterwards by calculating the weighted points, which were derived by taking the specific *ß* coefficient for each independent predictor, divided by the lowest *ß* coefficient of all the independent predictors, multiplied by two, and rounded to the nearest integer [20].

The performance of the modified A-DIVA scale was determined in terms of discrimination and calibration. The discriminative acquisition of the modified A-DIVA scale was performed with a receiver operating characteristics (ROC) analysis using the area under the curve (AUC) and its 95% CI [20,21]. Calibration of the scale was assessed by the Hosmer-Lemeshow goodness-of-fit test, of which the statistics were compared with a *χ*^2^ distribution and a *p* value was recorded [22,23,24]. Internal consistency measures whether several items that propose to measure the same general construct produce similar scores, and is measured with Cronbach’s alpha, a statistic calculated from the pairwise correlations between items [14,20,25]. A good Cronbach’s *α* should range from 0.7 to 0.9, whereas a Cronbach’s *α* lower than 0.7 may indicate a low degree of homogeneity and a value higher than 0.9 may imply item redundancy [25]. Generalizability and usability of the modified A-DIVA scale were determined by visually comparing the area under the ROC curves and the results of the Hosmer-Lemeshow tests of all units in which participants were included and measurements were performed [14,20]. Each participant received an additive risk score based on the sum of the points of each predictor. Results of this additive score were used to define three risk groups (low, moderate, and high risk) [11].

This multivariate prediction scale was reported according to the TRIPOD (Transparent Reporting of a multivariable prediction model for Individual Prognosis Or Diagnosis) Statement and according to the guidelines for reporting observational studies as described in the STROBE (Strengthening the Reporting of Observational Studies in Epidemiology) Statement [18,26]. A *p* value less than 0.05 was considered statistically significant throughout the study. SPSS, version 25.0 (SPSS Inc., Chicago, IL, USA) was used for all statistical analysis.

## 3. Results

A convenient sample of 3689 participants were recruited, of which 102 were excluded for data analyses because of data violation (84 participants) or an inability to provide informed consent (18 participants). A total of 3587 participants were included in this study, divided into a development cohort that consisted of 1255 participants, whereas the other 2332 participant were included in the evaluation cohort. All participants were in stable hemodynamic conditions. The demographics and baseline characteristics of the included participants are represented in Table 1 for both cohort separately. 

The first attempt success rate of the total cohort was 81% (2923 participants). Two attempts were needed in 425 participants (12%), whereas 138 participants (4%) needed 3 attempts, 33 participants (1%) needed 4 attempts, and 68 participants (2%) needed 5 punctures or more to create an intravenous access. Altogether, 4676 punctures were performed to create an intravenous access throughout this study. Data regarding the procedure intravenous cannulation were as shown in Table 2, in which a distinction is made between the group of patients with a successful first attempt, and those with a failed first attempt of peripheral intravenous cannulation.

A univariate regression analysis was performed with all 28 measured and registered predictors for a difficult intravenous access. As a result of this analysis, 21 variables were included in the multivariate logistic regression analysis (gender, age, weight, length, ASA physical status, participants’ race, active smoking, cardiac diseases, vascular diseases, chemotherapy treatment, haematological status, hypovolemia, side of cannulation, place of cannulation, cannulation on the dominant side, size of the intravenous catheter, if it was difficult to achieve an intravenous access in the past, practitioner’s expectation of difficult intravenous cannulation, whether or not a suitable vein could be identified by palpating and/or visualizing the extremity, and diameter of the cannulated vein). Five variables were included in the final prediction model, namely: a history of a difficult intravenous access (OR 2.7, 95% CI 1.6 to 4.4); a difficult intravenous access as expected by the practitioner prior to intravenous cannulation (OR 2.6, 95% CI 1.6 to 4.0); the inability to detect a dilated vein by palpating the extremity (OR 4.8, 95% CI 2.5 to 8.1); the inability to detect a dilated vein by visualization of the extremity (OR 5.9, 95% CI 2.5 to 10.1); and a diameter of the target vein less than 3 millimeters (OR 3.5, 95% CI 2.7 to 4.4), after backward elimination of factors without a significant relation with the outcome of interest based on the Wald statistic and P value (Table 3). The additive A-DIVA scale was created based on the *ß* coefficients of the multivariate logistic analysis, as represented in Table 4. All factors included in the A-DIVA scale had a value for each additive risk factor rounded to 1. The scores for existing risk factors represented an approximate percentage of a predicted difficult intravenous access for each participant. 

**Figure 1:**
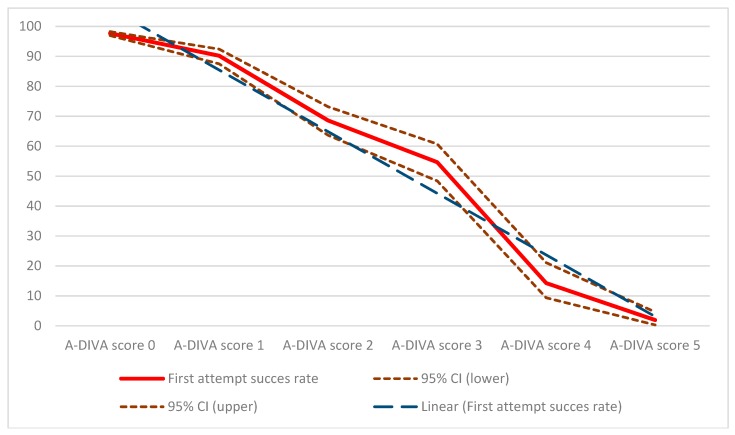
First attempt success rates by the depending A-DIVA (Adult Difficult Intra Venous Access) score. Solid line (red) = first attempt success rate. Dashed line (brown) = 95% confidence interval, divided into a lower and upper bound. Striped line (blue) = linear trend line of the first attempt success rates.

Based on this additive scale participants were classified into either a low, moderate, or high-risk group, depending on the existence of risk factors in the individual participant. The low risk group (A-DIVA score 0 or 1) included 2619 participants, of which 103 participants (4%) suffered from a failed first attempt. The moderate risk group (A-DIVA score 2 or 3) consisted of 610 participants with a failed first attempt in 226 participants (37%), and 358 participants were included in the high-risk group (A-DIVA score 4 or 5) with a failed first attempt observed in 335 participants (94%). Participants in the low risk group had a relative risk for a failed first attempt of 0.07 (95% CI 0.06 to 0.08), whereas participants in the moderate and high-risk group had a relative risk of 2.52 (95% CI 2.20 to 2.88) and 8.97 (95% CI 8.08 to 9.96) respectively. Participants with an A-DIVA score of 0 (none of the items on the scale were applicable to the participant), had a first attempt success rate of 98% (1999 of 2046 participants). To continue, first attempt success rates of 90% (517 of 573 participants), 69% (249 of 363 participants), 55% (135 of 247 participants), 14% (20 of 140 participants), and 2% (3 of 195 participants) were observed in participants with respectively an A-DIVA score of 1, 2, 3, 4, and 5 (which corresponds with the number of items that were scored on the scale), as shown in Figure 1.

The area under the ROC curve was 96% (SE (Standard Error) 0.002) for the development cohort, and 97% (SE 0.003) for the evaluation cohort. Goodness of fit of the additive A-DIVA scale in the evaluation cohort, tested with the Hosmer-Lemeshow statistic, resulted in a *χ*^2^ value of 13.57 (*p* = 0.104). In the development cohort, on the other hand, was a Hosmer-Lemeshow goodness of fit *χ*^2^ value detected of 15.58 (*p* = 0.086). Reliability analysis resulted in a Cronbach’s *α* of 0.78, with an intraclass correlation coefficient of 0.78 (95% CI 0.77 to 0.79, *p* < 0.001). Table 5 represents the results of the independent analyses for the generalizability and usability of the modified A-DIVA scale in terms of discrimination and calibration, after testing the A-DIVA scale in all units separately. 

A practitioner’s expectation of a difficult intravenous access prior to the procedure correlated with the outcome of a failed first attempt upon intravenous cannulation (Spearman’s Rho correlation coefficient *ρ* = 0.68, *p* < 0.001). A difficult intravenous access was expected in 711 participants (20%), of which 483 participants (68%) had a failed first attempt (*χ*^2^ = 1591.75, *df* = 1, *p* < 0.001). On the other hand, 118 (4%) of the 2876 participants without an expected difficult peripheral intravenous cannulation suffered from a failed first attempt. Factors determining whether a difficult intravenous access was expected by the practitioner were a known history of a difficult intravenous access (*ρ* = 0.43, *p* < 0.001), a participant’s body mass index (*ρ* = 0.06, *p* < 0.001), treatment with chemotherapy in a participants history (*ρ* = 0.10, *p* < 0.001), the diameter of the target vein after applying venodilation (*ρ* = 0.33, *p* < 0.001), diabetes mellitus (*ρ* = 0.08, *p* < 0.001), and the inability to detect a dilated vein on the upper extremity by either palpation (*ρ* = 0.45, *p* < 0.001) or visualization (*ρ* = 0.47, *p* < 0.001). Remarkably, however, not all of these factors were included in the A-DIVA scale based on the results of the logistic regression analyses, nor had they a positive correlation with the outcome of a failed first attempt of peripheral intravenous cannulation. 

## 4. Discussion

A five-variable additive A-DIVA scale was created, based on variables that affect the outcome of peripheral intravenous cannulation on the first attempt, including a known history of a difficult intravenous access, an expected difficult intravenous access by the practitioner prior to intravenous cannulation, the inability to detect a dilated vein by palpation and/or visualization of the extremity, and a diameter of the target vein less than 3 millimeters. A patient’s individual score on the A-DIVA scale will predict the likelihood of failed peripheral intravenous catheter placement. A higher score on the A-DIVA scale indicates a higher risk of the presence of a difficult intravenous access.

No consensus has been reached throughout recent publications reporting about failure upon peripheral intravenous cannulations and the factors influencing this outcome [5,6,27,28,29,30,31,32,33]. Reported first attempt success rates of peripheral intravenous cannulation varied from 51% to as high as 89%, which corresponds to the 81% first attempt success rate of peripheral intravenous cannulation found in this study [6,28,29,30]. Notwithstanding, this means that two out of ten patients will suffer from a failed attempt of intravenous cannulation. Factors often mentioned as risk factors for a difficult intravenous access or a failed first attempt throughout different studies included female gender, obesity, a history of treatment with chemotherapy, veins with many valves, sickle cell disease, burns, intravenous drug abuse, and diabetes mellitus [27,28,30,34,35,36,37,38]. Nevertheless, factors about a participant’s baseline demographics, social behavior, and medical history did not affect the outcome of interest as a result of the current study. A lack of visible and/or palpable veins were important risk factors for a failed first attempt as reported in the publications by Piredda and colleagues and Carr and colleagues. This is in line with the findings in this study [30,35]. Likewise, a smaller diameter of the target vein was reported as a risk factor for failure upon the first attempt of intravenous cannulation in previous publications [7,11]. Bensghir and colleagues reported an increased rate of failure upon the first attempt of cannulation when the intravenous catheter was inserted by nurses and physicians in training, although no differences between the practitioners on the success rates were observed in the present study [28].

Another important risk factor for a failed first attempt was an expected difficult intravenous access by the practitioner prior to intravenous cannulation as determined based on its own experience. Although this seems trivial, previous studies did not focus on a practitioner’s expectation of a difficult intravenous access. In contrast, recent publications denoted the factors underlying a practitioner’s expectation of a difficult intravenous access as absolute risk factors for failure of peripheral intravenous cannulation [28,30,34,35,36,37,38,39]. Despite having the smallest odds ratio as a result of the multivariate logistic analysis, which is in line with the results as shown in Table 2, this study has indeed shown that an expected difficult intravenous access by the practitioner more often resulted in a failed attempt of peripheral intravenous cannulation. Gut-feelings and expectations by practitioners regarding an outcome of interest must be taken seriously in healthcare, especially since the sense of alarm and the sense of reassurance are well-developed concepts in experienced caregivers [40,41,42]. Despite practitioners having a well-developed gut-feeling, it is assumed that a practitioner should be trained and experienced in peripheral intravenous cannulation before he can decide whether or not a difficult intravenous access can be expected. 

It is not always obvious which patients are likely to suffer from a failed first attempt of peripheral intravenous cannulation based on a difficult intravenous access, and thus unidimensional scales to classify those patients at risk should prospectively be created and used in daily practice. A patient can be classified as being at risk based on clearly defined cut-off points as determined on patient characteristics, as in the A-DIVA scale. The A-DIVA scale as created in the current study is a modified version of the previous developed A-DIVA scale, with the purpose to create a generalizable scale [11]. Recently, an Enhanced Adult DIVA (EA-DIVA) score was created by Civetta and colleagues [43]. This eight-item predictive scale was developed in a preoperative setting including surgical patients, although this EA-DIVA scale was merely validated internally. Another scale to identify patients at risk for a difficult intravenous access is the pediatric DIVA scale by Yen and colleagues [44]. Despite the fact that this scale passed both the internal and external validation processes, is it not applicable to the adult population [11,44,45].

Prospective identification of factors and patients at high risk for failure of peripheral intravenous cannulation creates a possibility to apply additional techniques in an earlier timeframe, possibly resulting in effective and efficient use of those techniques [6]. The application of additional, newly-developed techniques, such as point-of-care ultrasound, can be indicated for those patients at risk based on the A-DIVA scale. In analogy of previous results, the use of ultrasound seems to be beneficial in patients at high and moderate risk for a difficult intravenous access [6]. First attempt success rate only improved after comprehensive training of healthcare providers [46,47,48]. Therefore, it remains debatable whether ultrasound should be considered early if the vessel cannot be seen directly or palpated and peripheral venous cannulation proves to be difficult, as recommended by various guidelines [8]. In our opinion, efficient use of ultrasound can by indicated on the A-DIVA scale.

Further research should focus on the impact of the use of A-DIVA scale in clinical practice. We envisage that the A-DIVA scale can be an effective and efficient tool to guide usage of additional modern techniques for intravenous cannulation. A further research project should focus on the influence of the A-DIVA scale on success rates of peripheral intravenous cannulation in the different risk populations, patient comfort, and cost reductions as a result of an increased first attempt success rate. Analysis of the mechanisms underlying the persistent high rate of failure upon peripheral intravenous cannulation also reveals opportunities for improvement [5]. Gathering knowledge about the origin of failure of peripheral intravenous cannulation and risk classification as represented in this study will possibly lead to an acceptable rate of success on the first attempt.

### Limitations

Although this study was based on a large sample of participants in both the derivation and evaluation cohort and its multicenter design, are there some limitations to acknowledge. At first the study population consisted largely of surgical patients recruited from a preoperative setting, in contrast to the relatively smaller cohort of participants that were included from the labor ward and emergency department. The vast majority of participants were included from a preoperative holding department in the surgical theatre complex, which is potentially a different population compared with acute care patients in an emergency department, or patients that were presumably healthier with less comorbidity as were those in the labor ward. Besides, the population of surgical patients included both participants taken from an inpatient and outpatients setting, as well as patients for elective planned surgery, and patients for unplanned acute surgical procedures. Nevertheless, the multicenter design ensured recruitment of a wide range of patients from different hospitals, making translation of the results to the total hospitalized population feasible. Secondly some of the included factors were of a qualitative or subject nature, and therefore free for interpretation by the depending practitioner, for example, the participant’s skin shade or whether or not a vein could be detected by palpating or visualizing the extremity for instance. Inter-observer variability may have affected the results of measurements of the included risk factors. Inter-observer variability indicates the extent to which different observers reach the same judgment when performing the same measurement and indicates how sensitive the measurements are for the person who performs the measurements. Thirdly, the current study was set up with an observational design. In contrast to randomized trials, the method of including participants in a cross-sectional study creates a risk for selection bias. In the present study every patient requiring insertion of an intravenous catheter was included, in order to minimize the risk of selection bias. In addition, this current study was carried out according to the STROBE statement [26,49]. The influence of recall or information bias was thought to be minimal due to the prospective design [49]. Confounding bias is present when an event of interest is strongly associated with another event or exposure related to the outcome and could be an important limitation of observational studies as well, although the risk for confounding was taken into account during the analysis of the data [50].

## 5. Conclusions

In conclusion, the five-variable additive A-DIVA scale, as created in this study, is a reliable and generalizable predictive scale to identify patients at risk of a difficult intravenous access. The A-DIVA scale is based on factors including a known history of a difficult intravenous access, an expected difficult intravenous access by the practitioner prior to intravenous cannulation, the inability to detect a dilated vein by palpation and/or visualization of the extremity, and a diameter of the target vein less than 3 millimeters. The A-DIVA scale is validated both internally and externally as a result of this study. The A-DIVA scale can therefore be used in clinical practice.

## Figures and Tables

**Table 1:** Demographics and baseline characteristics of the included participants.

Variable	Category	Development Cohort (N = 1255)	Evaluation Cohort (N = 2332)
Gender	Male Female	598 (48%) 657 (52%)	1052 (45%) 1280 (55%)
Age	Years	55 (17)	59 (18)
Weight	Kilograms	83 (22)	81 (20)
Length	Centimeters	171 (10)	171 (11)
ASA classification	ASA 1 ASA 2 ASA 3 ASA 4	312 (25%) 581 (46%) 355 (28%) 7 (1%)	549 (24%) 1356 (58%) 362 (16%) 65 (2%)
Race	Asian Caucasian Mediterranean and Arabic Afro-European	0 (0%) 1119 (89%) 111 (9%) 25 (2%)	215 (9%) 1866 (80%) 168 (7%) 83 (4%)
Dominant side	Left Right	120 (10%) 1135 (90%)	167 (7%) 2165 (93%)

Demographic and baseline characteristics are represented as mean (standard deviation) for continuous data or as frequencies (percentages) for categorical data. ASA classification = American Society of Anesthesiology classification.

**Table 2:** Data representing the procedure of peripheral intravenous cannulation.

Variable	Category	Successful First Attempt (N = 2923)	Unsuccessful First Attempt (N = 664)
Number of attempts		1 (0)	2 (1)
Side of cannulation	Left Right	1549 (53%) 1374 (47%)	412 (62%) 252 (38%)
Place of cannulation	Dorsum of the hand Lower arm Elbow crease Upper arm	1900 (65%) 702 (24%) 292 (10%) 29 (1%)	378 (57%) 179 (27%) 100 (15%) 7 (1%)
Size of the catheter	22 gauge 20 gauge 18 gauge 16 gauge 14 gauge	117 (4%) 1491 (51%) 1140 (39%) 146 (5%) 29 (1%)	73 (11%) 391 (59%) 153 (23%) 27 (4%) 20 (3%)
History of difficult intravenous cannulation	Yes No	409 (14%) 2514 (86%)	412 (62%) 252 (38%)
Practitioners expectation of difficult intravenous access	Yes No	234 (8%) 2689 (92%)	266 (40%) 398 (60%)
Palpable vein after tourniquet placement	Yes No	2748 (94%) 175 (6%)	219 (33%) 445 (67%)
Visible vein after tourniquet placement	Yes No	2718 (93%) 205 (7%)	139 (21%) 525 (79%)
Both palpable and visible vein after tourniquet placement	Yes No	2835 (97%) 88 (3%)	286 (43%) 378 (57%)
Diameter of the vein after tourniquet placement	Millimeters	3 (1)	2 (1)
Practitioner	Physician Nurse	234 (8%) 2689 (92%)	73 (11%) 591 (89%)
Pain score on a verbal numeric rating scale	0–10	2 (2)	5 (3)

Data regarding the procedure of peripheral intravenous cannulation was represented as median (interquartile range) for continuous data or as frequencies (percentages) for categorical data.

**Table 3:** Multivariate regression analyses.

Factor	*ß*	SE	*p* Value	Odds Ratio	95% CI
History of a difficult intravenous cannulation	0.976	0.180	<0.001	2.7	1.6 to 4.4
Practitioner’s expectation of a difficult intravenous access	0.936	0.191	<0.001	2.6	1.6 to 4.0
No palpable vein after tourniquet placement	1.670	0.187	<0.001	4.8	2.5 to 8.1
No visible vein after tourniquet placement	1.879	0.192	<0.001	5.9	2.5 to 10.1
Diameter of the vein less than 3 millimeters after tourniquet placement	1.247	0.094	<0.001	3.5	2.7 to 4.4

Constant *ß* 8.950, SE 0.543, *p* < 0.001. SE = Standard Error. CI = Confidence Interval.

**Table 4:** The additive A-DIVA scale.

Factor	Score
Is there a known history of a difficult intravenous access?	1
Do you expect a failed first attempt or a difficult intravenous access?	1
Is there an inability to identify a dilated vein by palpating the upper extremity?	1
Is there an inability to identify a dilated vein by visualizing the upper extremity?	1
Has the largest dilated vein a diameter less than 3 millimeters?	1

The additive A-DIVA scale is represented as an additive scoring system to calculate the predicted risk for an individual patient; the scores for existing risk factors are added to give an approximate estimation of a difficult intravenous access. Scores are added after answering a question with “yes”.

**Table 5:** Comparison of the level of performance of the modified A-DIVA scale in the different included units.

Unit (Hospital and Department)	Success Rate on the First Attempt	AUC of the ROC Curve	SE of the AUC	Hosmer-Lemeshow *χ*^2^	Hosmer-Lemeshow *p* Value
Overall cohort (N = 3587)	83%	97%	0.003	15.58	0.086
Unit 1 ^a^ (N = 1212)	85%	96%	0.002	10.04	0.190
Unit 2 ^a^ (N = 848)	80%	98%	0.003	2.94	0.201
Unit 3 ^a^ (N = 598)	86%	98%	0.007	3.00	0.223
Unit 4 ^a^ (N = 433)	81%	97%	0.009	4.49	0.344
Unit 5 ^a^ (N = 230)	83%	96%	0.012	11.34	0.096
Unit 6 ^b^ (N = 162)	84%	93%	0.011	9.82	0.076
Unit 7 ^c^ (N = 104)	71%	77%	0.078	21.86	0.054

^a^ = Participants were recruited from a surgical department in the preoperative holding area of the theatre complex. ^b^ = Participants were recruited from an emergency department. ^c^ = Participants were recruited from a labor ward. AUC = Area Under the Curve. ROC = Receiving Operators Curve.
